# HDAC4 Does Not Act as a Protein Deacetylase in the Postnatal Murine Brain *In Vivo*

**DOI:** 10.1371/journal.pone.0080849

**Published:** 2013-11-22

**Authors:** Michal Mielcarek, Tamara Seredenina, Matthew P. Stokes, Georgina F. Osborne, Christian Landles, Linda Inuabasi, Sophie A. Franklin, Jeffrey C. Silva, Ruth Luthi-Carter, Vahri Beaumont, Gillian P. Bates

**Affiliations:** 1 Department of Medical and Molecular Genetics, King’s College London, London, United Kingdom; 2 Brain Mind Institute, Ecole Polytechnique Federale de Lausanne, Lausanne, Switzerland; 3 Cell Signaling Technology, Danvers, Massachusetts, United States of America; 4 CHDI Management Inc./CHDI Foundation Inc., Los Angeles, California, United States of America; Emory University, United States of America

## Abstract

Reversible protein acetylation provides a central mechanism for controlling gene expression and cellular signaling events. It is governed by the antagonistic commitment of two enzymes families: the histone acetyltransferases (HATs) and the histone deacetylases (HDACs). HDAC4, like its class IIa counterparts, is a potent transcriptional repressor through interactions with tissue specific transcription factors via its N-terminal domain. Whilst the lysine deacetylase activity of the class IIa HDACs is much less potent than that of the class I enzymes, HDAC4 has been reported to influence protein deacetylation through its interaction with HDAC3. To investigate the influence of HDAC4 on protein acetylation we employed the immunoaffinity-based AcetylScan proteomic method. We identified many proteins known to be modified by acetylation, but found that the absence of HDAC4 had no effect on the acetylation profile of the murine neonate brain. This is consistent with the biochemical data suggesting that HDAC4 may not function as a lysine deacetylase, but these *in vivo* data do not support the previous report showing that the enzymatic activity of HDAC3 might be modified by its interaction with HDAC4. To complement this work, we used Affymetrix arrays to investigate the effect of HDAC4 knock-out on the transcriptional profile of the postnatal murine brain. There was no effect on global transcription, consistent with the absence of a differential histone acetylation profile. Validation of the array data by Taq-man qPCR indicated that only *protamine 1* and *Igfbp6* mRNA levels were increased by more than one-fold and only *Calml4* was decreased. The lack of a major effect on the transcriptional profile is consistent with the cytoplasmic location of HDAC4 in the P3 murine brain.

## Introduction

The acetylation of specific lysine residues influences the activity of many proteins including histones and this process has been shown to be a central mechanism controlling gene expression and cell signaling events. There is an increasing body of evidence to suggest that chromatin structure and epigenetic regulation are major players in the pathology of many diseases including neurodegenerative disorders [1]. Reversible lysine acetylation is controlled by the antagonistic commitment of two enzymes families: the histone acetyltransferases (HATs) and the histone deacetylases (HDACs) [2]. The 18 human HDACs can be clustered into four different classes, based on their sequence homology to the yeast orthologus Rpd3, Hda1 and Sir2. The class I HDACs have high homology to Rpd3 and include HDAC1, -2, -3- and -8. Class II HDACs are homologous to Hda1 and are divided into two subclasses: IIa (HDAC4, -5, -7, -9) and IIb (HDAC6 and HDAC10). Class III HDACs have high homology to yeast Sir2 and comprise the sirtuins: SIRT 1-7. Finally class IV contains only HDAC11, which shares homology with both class I and II enzymes [2].

In comparison to the other classes of HDACs the class II enzymes display a number of unique features. Unlike the HDAC1 enzymes that are predominantly localised in nuclei, the class IIa enzymes shuttle between the nucleus and cytoplasm, a process that is controlled through the phosphorylation of specific serine residues within their N-terminal domains [3-5]. The class IIa HDACs are potent transcriptional repressors, a function that is mediated through the regulatory N-terminal domains that interact with tissue specific transcriptional factors [3], and is dependent upon their presence in the nucleus [4]. Finally, in contrast to the other HDACs, the C-terminal catalytic domain of the class IIa enzymes contains a histidine substitution of a critical tyrosine residue that has been shown to render them comparatively inactive as lysine deacetylases [6].

HDAC4 is highly expressed in the mouse brain as compared to the other class IIa enzymes [7] with the highest expression occurring during early postnatal life [8]. In various experimental models, it has been shown that the loss of HDAC4 can lead to neurodegeneration during the development of the retina [9] and cerebellum [10]. Moreover, partial loss of *Hdac4* in the mouse forebrain under the *CamkII* promoter, revealed impairments in hippocampal-depend learning and memory with a simultaneous increase in locomotor activity [11]. In the light of these findings, it was surprising that the selective deletion of *Hdac4* under the Thy1 or nestin promoters did not alter the gross morphology or cytoarchitecture of the brain and resulted in normal locomotor activity [12]. Similarly hippocampal depletion of HDAC4 *in vivo* abolished long-lasting stress-inducible behavioural changes and improved stress related learning and memory impairments in mice [13]. Finally, HDAC4 overexpression has been shown to accelerate the death of cerebellar granule and neurons [8,14,15] and rendered neurons more vulnerable to a H_2_0_2_ insult by inhibiting PPARγ activity (peroxisome proliferators-activated receptor γ)[16]. 

To further explore the biological function of HDAC4 in brain, we have investigated whether loss of HDAC4 in the postnatal mouse brain causes global changes in the acetylation status of various proteins and/or results in major changes to transcriptional profiles *in vivo*.

## Results

We have employed a genetic approach to investigate the extent to which HDAC4 contributes to global changes in protein acetylation in brain. *Hdac4* knock-out (KO) mice are viable until early postnatal life [17], therefore, it was possible to compare the pattern of protein acetylation between wild type (WT) and HDAC4 null brains from neonates at three days of age (P3), an age at which HDAC4 is highly expressed [8].

We used Cell Signalling Technology’s AcetylScan proteomics platform [18-21] to identify and quantify differences in acetylation between WT and *Hdac4*KO mouse brains. The AcetylScan method combines the isolation of acetylated peptides from protease digested protein extracts using a proprietary immunoaffinity purification method followed by the identification and quantitation of peptides by liquid chromatography with tandem mass spectrometry (LC-MS/MS) on an LTQ-Orbitrap mass spectrometer. Chromatographic peak apex intensities of peptide ions in each sample are derived from their corresponding extracted ion chromatograms. Label-free quantitation is performed by comparing peak intensities of the same peptide ion in each sample to generate their corresponding fold-changes. 

**Figure 1:**
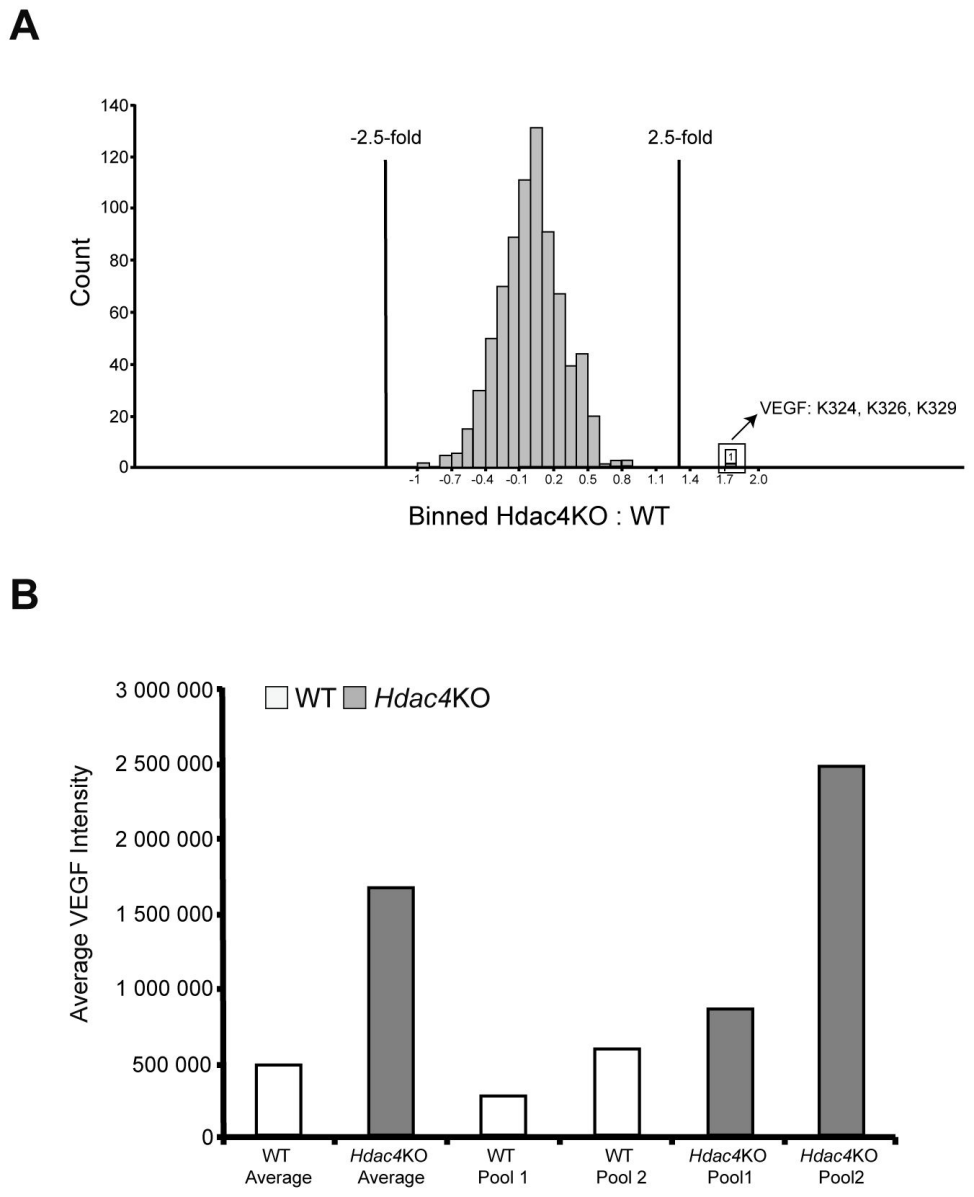
Absence of HDAC4 causes only minor changes to the neonate brain acetylome. (A) Histogram showing normalized log_2_ ratios between HDAC4 KO and WT brains for all peptides identified. The VEGF peptide with a >2.5-fold change is highlighted. (B) Relative abundance for VEGF K324, 326, 329 acetylated peptide across all samples (control and HDAC4 KO pool1 and pool 2).

The Acetylscan method was performed on two independent pools of 2-3 brains (0.5 g) for each of the *Hdac4*KO and WT genotypes (pool 1 or pool 2) and each pool was run in duplicate. 4,247 redundant peptide assignments containing acetylated lysines from 1,164 non-redundant proteins were identified, including peptide sequences from proteins with known acetylation signatures such as histones, tubulin and many others (Table S1). Our analysis focussed on those peptides that had a maximum intensity of 10-fold above background (200,000 intensity with a background of 20,000), and a cut-off of greater than 2.5-fold, to determine whether peptides were differentially acetylated between genotypes [18,22]. The quantitative data was reproducible, with biological (inter-pool) %CV’s of 17.2% to 22.5%, and analytical (intra-pool) %CV’s of 10.9% to 12.5% (see Table S1). The fold change measurements for all peptides are displayed in the histogram in Figure 1A, with the ±2.5-fold change cut-offs delineated. The histone-derived peptides were well above the intensity cut-off and are summarised in Table S1, and the fold changes indicate that there was no difference in the degree of histone acetylation between WT and *Hdac4*KO brains. There was only one peptide that could be considered to approach our cut-off criteria in both biological replicates (Figure 1A). This differential acetylation signature was identified for VEGF (vascular endothelial growth factor A isoform 1 precursor) triply acetylated at Lys324, Lys326, and Lys329. The mean raw intensity values for the VEGF affinity purified peptides are shown in Figure 1B. Other acetylated peptides, including those from DYN1, ATAT1, RAI1, Beta-s, and GPB1/2 showed a change in abundance between the WT and *Hdac*4KO brains, however, these changes were not consistent across both brain pools (Table S1). Therefore, we were able to find very little evidence to support a change in the acetylation status of proteins caused by the absence of HDAC4, with lysine 324, 326, and 329 in VEGF providing the only strong candidate. 

As we did not observe changes in histone acetylation in response to HDAC4 depletion, we would predict that loss of HDAC4 would not result in global transcriptional dysregulation. However, we would expect some transcriptional changes as HDAC4 is known to repress transcription through the interaction of specific transcription factors with its N-terminal domain. To investigate this further, we performed Affymetrix array profiling on WT and *Hdac4*KO brains from P3 neonates. RNA was prepared from five brains per gender per genotype (n = 10/genotype) and profiled using the mouse genome 430_2 arrays. Gene expression changes were filtered for a false discovery rate (FDR *p*<0.05) and the number of genes for which the level of expression differed between the genotpes by more than 20% and 30% is listed in Table 1. The microarray data have been deposited in the NCBI GEO database with the accesssion number GSE 4938. 

**Table 1:** Transcriptional changes between WT and *Hdac4*KO P3 brains as detected by Affymetrix array profiling.

Probe sets	More than 20% change	More than 30% change
Downregulated	363	69
Upregulated	109	22

Affymetrix arrays were used to determine the effect of *Hdac4* knock-out on the transcription profile in the postnatal P3 brain (n=10 per genotype). The number of genes that were significantly altered between genotypes with a fold-change of >30% or >20% for each pairwise comparison is noted. Statistical significance was determined after FDR-correction at a stringency of *p*≤0.05.

**Figure 2:**
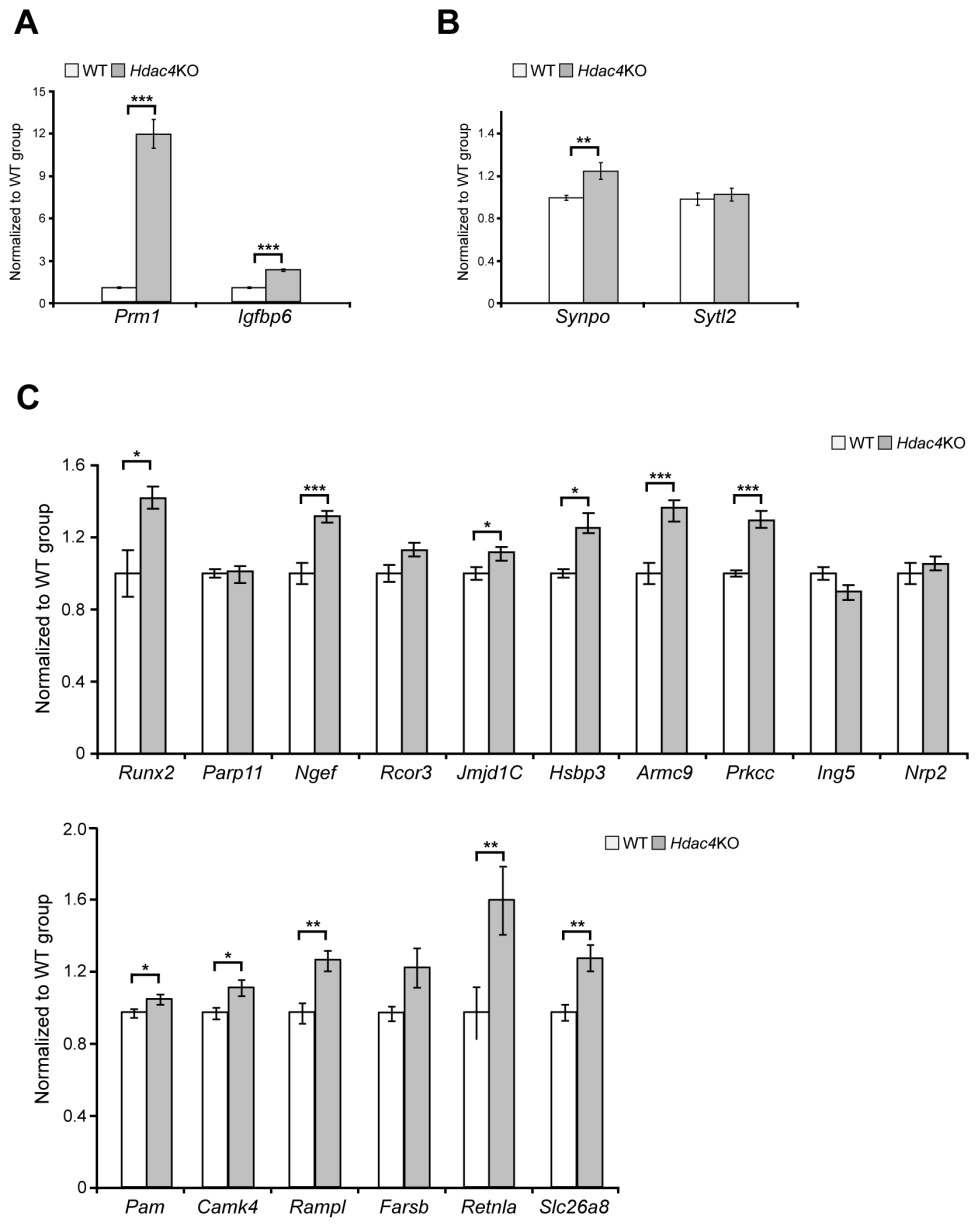
Validation of the genes predicted to be upregulated in *Hdac4* knock-out P3 brains. (A) *Prm1* (protamine 1) and *Igbp6* (insulin-growth factor binding protein 6) transcript levels were significantly upregulated in *Hdac4*KO P3 brains by 14-fold and 3-fold respectively. (B) *Synpo* (Synaptopodin) was modestly upregulated in *Hdac4*KO P3 brains whereas *Sytl2* (synaptotagmin like 2 protein) was unchanged. (C) Runx2 (runt-related transcription factor 2), *Ngef* (neuronal guanine nucleotide exchange factor), *Jmjd1C* (jumonji domain containing 1C), *Hsbp3* (heat shock protein 3), *Armc9* (armadilo repeat containing protein 9), *Prkcc* (protein kinase C gamma), Pam (peptidylglycine alpha-amidating monooxygenase), CamkIV (calcium /calmodulin-dependent protein kinase IV), *Ramp1* (receptor (calcitonin) activity modifying protein 1), *Retnlα* (resistin like alpha protein), *Slc26α8* (solute carrier family 26, member 8) were modestly upregulated in *Hdac4*KO P3 brains whereas *Parp11* (poly(ADP-ribose) polymerase member 11), *Rcor3* (REST corepresor 3), Ing5 (inhibitor of growth family 5) Nrp2 (neuropilin 2) and *Farsb* (phenylalanyl-tRNA synthetase, beta subunit) were unchanged. All mRNA expression levels were assessed by Taqman qPCR and presented as a relative expression ratio to the geometric mean of three housekeeping genes *Atp5b*, *Canx*, *Rpl13α*. Error bars are S.E.M (n>7). *p<0.05, **p<0.01, ***p<0.001.

**Figure 3:**
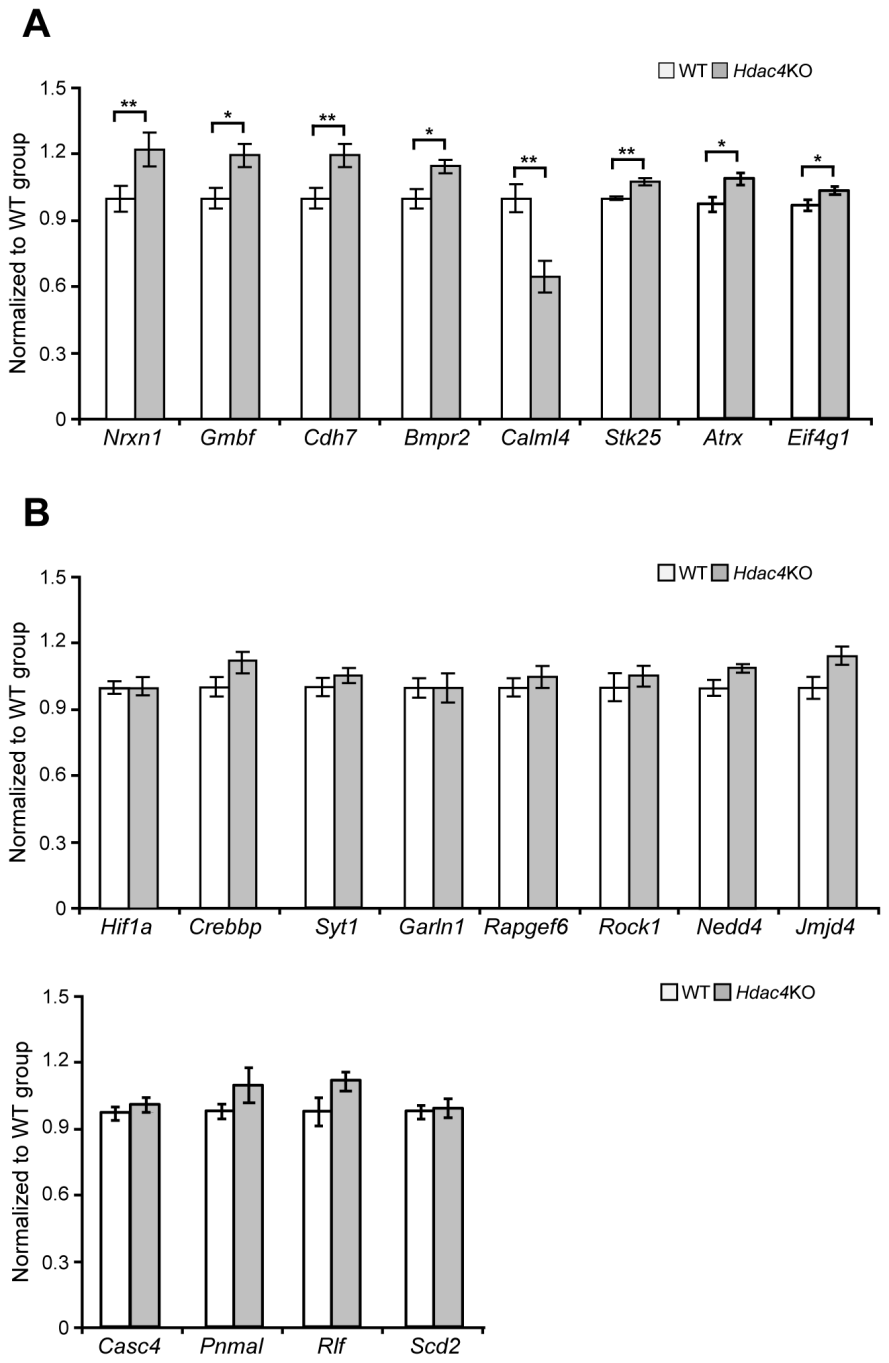
Validation of the genes predicted to be downregulated in *Hdac4* knock-out P3 brains. (A) *Nrxn1* (neurexin1), *Gmbf* (glia maturation factor beta), Cdh7 (cadherin 7), *Bmpr2* (bone morphogenic protein receptor, type II), Stk25 (serine/threonine kinase 25), *Atrx* (alpha thalassemia/mental retardation syndrome X-linked homolog) and *Eif4g1* (eukaryotic translation initiation factor 4, gamma 1) were modestly upregulated in *Hdac4*KO P3 brains. *Calml4* (calmodulin-like 4) was the only gene that was downregulated (B) The expression level of Hif1α (hypoxia inducible factor 1 alpha subunit), *Crebbp* (CREB-binding protein), Syt1 (synaptotagemin 1) *Garln1* (GTPase activating RANGAP domain like 1 protein), *Rapgef6* (Rap guanine nucleotide exchange factor GEF6), Rock1 (Rho-associated coiled-coil containing protein kinase 1), *Nedd4* (NEDD4 binding protein), *Jmjd4* (jumonji containing protein 4), *Casc4* (cancer susceptibility candidate 4), *Pnmal* (PNMA-like 2), *Rlf* (rearranged L-myc fusion sequence) and Scd2 (stearoyl-Coenzyme A desaturase 2) did not change between *Hdac4*KO and WT P3 brains. All mRNA expression levels were assessed by Taqman qPCR and presented as a relative expression ratio to the geometric mean of three housekeeping genes *Atp5b*, *Canx*, *Rpl13α*. Error bars are S.E.M (n>7). *p<0.05, **p<0.01.

To validate these data we performed Taqman quantitiative real-time PCR (qPCR) (n = 10/genotype) for 20 upregulated and 20 downreguled genes selected on the basis of their fold-change, FDR-corrected *p*-value and biological function. Of the upregulated genes: *Prm1* (protamine 1) was increased by 12-fold, and *Igfbp6* (insulin-growth factor binding protein 6) by more than 2-fold (Figure 2A). Of the synaptic proteins, there was a minor (20%) but significant increase in *Synpo* (synaptopodin) but not *Sytl2* (synaptotagmin like 2 protein) (Figure 2B). Of the remaining 16 upregulated transcripts, there was a small increase (up to 50%) for 11 genes (Figure 2C). We performed a similar validation for the downregulated transcript levels (Figure 3). Surprisingly, a reduction in expression could only be validated for *Calml4* (calmodulin-like 4) which was decreased by approximately 40% of WT (Figure 3A). In contrast, we observed a minor but significant increase for *Nrxn1* (neurexin1), *Gbmf* (Glia maturation factor beta), *Cdh7* (cadherin 7), *Bmpr2* (bone morphogenic protein receptor, type II), *Stk25* (serine/threonine kinase 25), *Atrx* (alpha thalassemia/mental retardation syndrome X-linked homolog) and (*Eif4g1* eukaryotic translation initiation factor 4, gamma 1). A change in expression for the remaining 12 genes was not detected (Figure 3B). 

**Figure 4:**
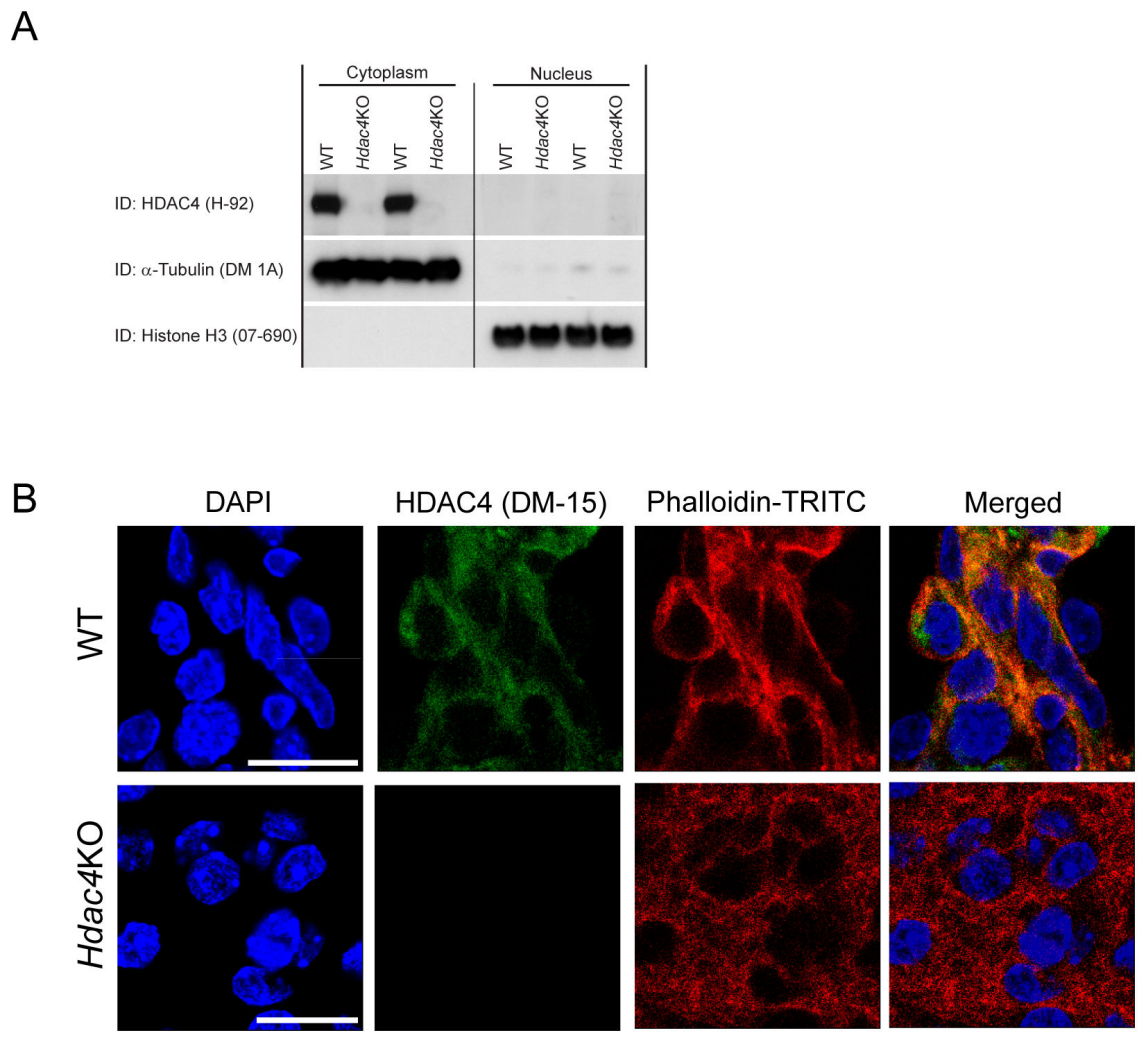
HDAC4 is localised to the cytoplasm in the P3 brain. (A) Western blot showing that HDAC4 is localised exclusively to the cytoplasm and absent in *Hdac4*KO brains. (B) Confocal images demonstrating that HDAC4 is localised to the cytoplasm. Scale bar = 25 μm.

These data prompted us to investigate the localisation of the steady-state levels of HDAC4 in the brains of P3 neonates which was found to be cytoplasmic by both western blotting and immunohistochemistry (Figure 4). 

## Discussion

During cell homeostasis, HATs and HDACs are maintained in a highly balanced state to efficiently regulate cell equilibrium leading to normal neurophysiological outputs [23]. There is accumulating evidence that altered chromatin plasticity and protein acetylation are involved in many neuropathological processes an as well in ageing [24]. HDAC4, like the other class IIa HDAC members, shuttles between the nucleus and the cytoplasm in response to specific stimuli [25,26]. It is expressed abundantly in the adult mouse brain [27] and localizes predominantly to the cytoplasm of most neurons and dendritic spines [7]. To date, the biological role of HDAC4 has been linked either to its possible deacetylase activity or to its potency to repress transcription factors, specifically myocyte enhancing factors (MEFs) [2]. The aim of this study was to evaluate the potential of HDAC4 as a protein deacetylase or transcriptional repressor in the murine brain during early postnatal life. To this end, we employed two unbiased screens: the AcetylScan proteomics platform and Affymetrix transcriptomic arrays. 

If HDAC4 acts as a lysine deacetylase, either directly or indirectly, we reasoned that the absence of HDAC4 would be expected to result in a perturbation in the acetylome. To test this hypothesis, we applied the AcetylScan platform to determine whether lysine acetylation was increased in *Hdac4* knockout brains as compared to WT, at 3 days of age, when HDAC4 is highly expressed [8]. Peptides containing acetylated lysine residues that derive from proteins known to be modified were detected at high signal intensities e.g histones, tubulin, 14-3-3 isoforms tau and dystrophin (Table S1). We were only able to detect a possible *Hdac4* knock-out related increase in acetylation at lysines 324, 326 and 329 in VEGF (vascular endothelial growth factor A). This change in acetylated peptide abundance may have been caused indirectly, or, given that our analysis cannot distinguish between an increase in lysine acetylation and an increase in the expression level of a protein, may reflect an increase in VEGF levels. These data indicate that HDAC4 does not act as a global protein deacetylase *in vivo*. Whilst we cannot rule out that the absence of HDAC4 may alter the aceytylation of specific protein, our data are consistent with the demonstration that the ancestral substitution of a tyrosine residue for histidine in the catalytic domain of HDAC4, as well as the other class IIa HDACs, renders them inactive as lysine deacetylases [6,28]. It has also been proposed that the disruption of the interaction between HDAC4 and HDAC3 influences histone acetylation through the activation of the catalytic domain of HDAC3 [29]. However, given that histone acetylation was not increased in the *Hdac4K*O brains, our *in vivo* data do not support this hypothesis. 

To complement the AcetylScan study we used Affymetrix arrays to compare the transcriptional profiles of the *Hdac4*KO and WT P3 brains. The relative paucity of changes is consistent with our failure to detect global increases in histone acetylation. HDAC4 is known to inhibit transcription through its interaction with MEF2 [30] and comparison of our array data to the genome-wide expression profile of MEF2-dependent transcripts in brain [31] indicated that we were unable to detect changes in MEF2 dependent transcripts. We were also unable to reproduce changes in the expression of synaptic genes that were previously induced through the artificial relocation of HDAC4 to the nucleus [8]. These findings are consistent with our data showing that the steady-state levels of HDAC4 in the murine P3 brain are cytoplasmic. However, the array data were not completely devoid of changes in gene expression. We did detect a profound increase in the levels of *protamine 1* mRNA (> 12 fold) and a substantial increase in *Igfbp6* mRNA (>2 fold), the mechanisms for which are not clear. Out of the other top 26 deregulated transcripts, 12 were slightly increased (mostly less than 30%) and *Calml4* was the only transcript to be downregulated. A connection between these modestly dysregulated genes and the function of HDAC4 cannot be inferred from the literature. Our results are consistent with an absence of major pathological changes in the *Hdac4* knock-out murine postnatal brain at P3 [10]. Similarly, it was recently shown that the selective deletion of *Hdac4* under the control of the Thy1 or nestin promoters resulted in normal gross brain morphology and cytoarchitecture and as well in normal locomotor activity [12]. 

Taken together our data strongly suggest that HDAC4 is not involved in protein deacetylation. It is mainly localized to the cytoplasm in postnatal and adult murine brains [32], and consequently plays only a minor role as a transcriptional repressor in the postnatal murine brain *in vivo*.

## Materials and Methods

### Ethics statement

All experimental procedures performed on mice were conducted under a project licence from the Home Office and approved by the King's College London Ethical Review Process Committee.

### Mouse maintenance and breeding

The *Hdac4* knock-out colony was maintained by backcrossing heterozygous males to B6CBAF1/OlaHsd females (Harlan Olac, Bicester, UK). Homozygotes were generated by intercrossing as required. All animals had unlimited access to water and breeding chow (Special Diet Services, Witham, UK). Mice were subject to a 12 hour light/dark cycle. 

### Genotyping

Genomic DNA was isolated from an ear-punch. The *Hdac4* knock-out mice were genotyped by PCR. The primers for the WT allele were (CTTGTTGAGAACAAACTCCTGCAGCT and AGCCCTACACTAGTGTGTGTTACACA and for mutant allele were (AGCCCTACACTAGTGTGTGTTACACA and CCATGGATCCTGAGACTGGGG). PCR conditions were as follows: 4 min at 95°C, 35x (1 min at 95°C, 30 sec at 65°C, 1.5 min at 72°C) and a final extension for 10 min at 72°C as previously described [17].

### RNA extraction and Taqman real-time PCR expression analysis

Total RNA from whole postnatal brains at P3 was extracted with the mini-RNA kit accordingly to manufacturer instructions (Qiagen). The reverse transcription reaction (RT) was performed using MMLV superscript reverse transcriptase (Invitrogen) and random hexamers (Operon) as described elsewhere [33]. The final RT reaction was diluted 10-fold in nuclease free water (Sigma). All Taqman qPCR reactions were performed as described previously [33] using the Chromo4 Real-Time PCR Detector (BioRad). Estimation of mRNA copy number was determined in triplicate for each RNA sample by comparison to the geometric mean of three endogenous housekeeping genes (Primer Design) as described [34]. Primer and probe sets for gene of interest were purchased from Primer Design.

### Affymetrix gene expression arrays

For the Affymetrix arrays: biotinylated cRNAs were prepared from 200 ng total RNA using the GeneChip 3′ IVT Express Kit (Affymetrix) following the manufacturer’s instructions. cRNA (15 μg) was hybridized to GeneChip Mouse Genome 430 version 2.0 Arrays (Affymetrix) and processed, stained, and scanned according to the manufacturer’s recommendations. The quality of input RNAs and cRNAs was verified with the Bioanalyzer 2100 (Agilent Technologies) before use. Microarray quality control was performed using the software package provided on RACE [35]. Chips with a median normalized unscaled standard error greater than 1.05 were excluded. Affymetrix annotations (version 3.0) were used for probeset-to-gene assignments. 2-tailed *t*-test was performed to assess the differences in gene expression between groups for each genotype (WT n=10; *Hdac4*KO n=10). Corrections for multiple testing were performed using the false discovery rate (FDR) according to Benjamini and Hochberg [36] with a significance threshold of *p*<0.05.

### Sample Preparation and Mass Spectrometry

The extent of lysine acetylation was determined by Cell Signalling Technology using their Acetylscan platform. Whole mouse brains (two pools each of 2-3 whole brains to obtain the final weight of 0.5 g) were brought to 10 ml each with urea lysis buffer, homogenized twice for 20 seconds, sonicated at 15 W output power once for 25 seconds, and centrifuged for 15 min at 20,000 x g to remove insoluble material. The resulting “cleared” protein extracts were reduced and carboxamidomethylated. Total protein for each tissue type was normalized prior to trypsin digestion. Proteins were digested overnight with trypsin (Worthington). Peptides were separated from non-peptide material by solid-phase extraction with Sep-Pak C18 cartridges (Waters). Lyophilized peptides were redissolved in immunoaffinity purification buffer, and acetylated peptides were isolated using slurries of the appropriate immobilized motif antibody (CST # 9895). Peptides were eluted from antibody-resin into a total volume of 100 μl in 0.15% TFA. Eluted peptides were concentrated with Eppendorf PerfectPure C18 tips immediately prior to LC-MS analysis.

The samples from each of the pools were run in duplicate to generate analytical replicates and increase the number of MS/MS identifications from each sample. Peptides were loaded directly onto a 10 cm x 75 μm PicoFrit capillary column packed with Magic C18 AQ reversed-phase resin. The column was developed with a 90-min linear gradient of acetonitrile in 0.125% formic acid delivered at 280 nl/min. Tandem mass spectra were collected with an LTQ-Orbitrap hybrid mass spectrometer, a top 10 method, a dynamic exclusion repeat count of 1 and a repeat duration of 30 sec. MS spectra were collected in the Orbitrap component of the mass spectrometer, and MS/MS spectra were collected in the LTQ. MS/MS spectra were evaluated using SEQUEST 3G and the SORCERER 2 platform from Sage-N Research (v4.0, Milpitas CA) [37]. Samples were searched against the NCBI *mus musculus* database updated on 9/10/2010 with a 1X reverse database included for false discovery rate estimation. Peptide assignments were obtained using a 5% false positive discovery rate. Cysteine carboxamidomethylation was specified as a static modification, oxidation of methionine residues was allowed, and acetylation was allowed on lysine residues. Results were further filtered by mass error (–/+ 3 ppm) and the presence of an acetylated residue in the peptide. Labelled MS/MS spectra for all peptides identified in the study are provided as hyperlinks in the MS2 Spectrum Number columns of the Details Tab of Table S1.

### Label-Free Quantitation

Label-free quantitation was performed as previously described [18,22]. Changes in acetylated peptide levels were measured by taking the ratio of raw intensities between WT and *Hdac4*KO tissues, from pool 1 and pool 2 individually and pool 1 and pool 2 combined, with the control tissues as the reference (denominator). Log_2_ ratios were determined and the median log_2_ ratio was used to normalize the ratios for each binary comparison. Median offsets ranged from 0.07 (1.05-fold change offset) to 0.30 (1.23-fold change offset). Both raw and normalized fold changes for each comparison are shown in Table S1.

To make the quantification tables more complete, a proprietary computational program was used to search for acetylated peptide ions in the ion chromatogram files on the basis of their chromatographic retention times and their mass-to-charge (m/z) ratios for all acetylated peptides identified by MS/MS in at least one sample. The retention time window used was variable and based on the pattern seen in the extracted ion chromatogram files, and the m/z range used) ±3 ppm) was dependent on the mass error narrowing performed in a previous step. The computational program collected each peptide ion’s retention time, observed m/z ratio, and intensity. Peak intensity measurements for many peptide ions were manually reviewed in the ion chromatogram files. This eliminated the possibility that the automated process selected the wrong chromatographic peak from which to derive the corresponding intensity measurement. In cases where peak shapes were not consistent across all runs, peak apex intensities were substituted with integrated peak areas (A or H in Table S1) 

### Antibodies and western blotting

The primary antibodies used in this study were: HDAC4 (Santa Cruz H-92, 1:1,000), α-tubulin (Sigma DM 1A, 1:40,000) and histone H3 (Millipore 07-690, 1:40,000). The secondary antibodies were from DAKO: anti-mouse HRP (1:3,000), anti-rabbit HRP (1:3,000). Nuclear and cytoplasmic fractions were prepared as previously described [38], western blotted as described [39] and their purity was determined by immunoblotting with antibodies to anti-histone H3 and α-tubulin. 

### Immunohistochemistry and confocal microscopy

For immunohistochemical studies, brains were frozen in isopentane at -50°C and stored at -80°C until further analysis 10-15 μm sections were cut using a cryostat (Bright instruments), air dried and immersed in 4% PFA in PBS for 15 min and washed for 3x 5 min in 0.1% PBS-Triton X-100. Blocking was achieved by incubation with 5% BSA-C (Aurion) in 0.1% PBS-Triton X-100 for at least 30 min at RT. Immunolabelling with HDAC4 (Sigma DM-15, 1:100) was performed in 0.1% PBS-Triton X-100, 1% BSA-C overnight in a humidity box at 4°C. Sections were washed 3x in PBS, incubated for 60 min at RT in a dark box with the anti-rabbit (FITC Invitrogen 1:1000 in PBS), washed 3x in PBS, followed by 30 minutes incubation with phalloidin TRITC (Sigma, 1:100) and counterstained with DAPI (Invitrogen). Sections were mounted in Vectashield mounting medium (Vector Laboratories). Sections were examined using the Leica TCS SP4 laser scanning confocal microscope and analysed with Leica Application Suite (LAS) v5 (Leica Microsystems, Heidelberg, Germany).

### Statistical analysis

Unless otherwise stated, data were analysed with Microsoft Office Excel and Student's *t*-test (two tailed).

## Supporting Information

Table S1 **Summary data table of all acetylated peptides identified in the AcetylScan study.** The Details tab is a redundant list of all identifications with accompanying scoring metrics and links to labelled MS/MS spectra. The Summary tab is a non-redundant list of proteins/sites identified in the study with relative fold changes.
(XLSX)
